# Characterization of Volatile and Particulate Emissions from Desktop 3D Printers

**DOI:** 10.3390/s23249660

**Published:** 2023-12-06

**Authors:** Melissa Finnegan, Colleen Lee Thach, Shirin Khaki, Emma Markey, David J. O’Connor, Alan F. Smeaton, Aoife Morrin

**Affiliations:** 1 School of Chemical Sciences, National Centre for Sensor Research, Dublin City University, D09 DXA0 Dublin, Ireland; melissa.finnegan22@mail.dcu.ie (M.F.); shirin.khaki2@mail.dcu.ie (S.K.); david.x.oconnor@dcu.ie (D.J.O.); 2 Insight SFI Research Centre for Data Analytics, Dublin City University, D09 Y5N0 Dublin, Ireland; alan.smeaton@dcu.ie; 3 Department of Chemistry, The University of Kansas, Lawrence, KS 66046, USA; cthach@ku.edu; 4 School of Chemical Sciences, Dublin City University, D09 Y5N0 Dublin, Ireland; emma.markey5@mail.dcu.ie

**Keywords:** 3D printing, additive manufacturing, volatile organic compounds, particulate emissions

## Abstract

The rapid expansion of 3D printing technologies has led to increased utilization in various industries and has also become pervasive in the home environment. Although the benefits are well acknowledged, concerns have arisen regarding potential health and safety hazards associated with emissions of volatile organic compounds (VOCs) and particulates during the 3D printing process. The home environment is particularly hazardous given the lack of health and safety awareness of the typical home user. This study aims to assess the safety aspects of 3D printing of PLA and ABS filaments by investigating emissions of VOCs and particulates, characterizing their chemical and physical profiles, and evaluating potential health risks. Gas chromatography–mass spectrometry (GC–MS) was employed to profile VOC emissions, while a particle analyzer (WIBS) was used to quantify and characterize particulate emissions. Our research highlights that 3D printing processes release a wide range of VOCs, including straight and branched alkanes, benzenes, and aldehydes. Emission profiles depend on filament type but also, importantly, the brand of filament. The size, shape, and fluorescent characteristics of particle emissions were characterized for PLA-based printing emissions and found to vary depending on the filament employed. This is the first 3D printing study employing WIBS for particulate characterization, and distinct sizes and shape profiles that differ from other ambient WIBS studies were observed. The findings emphasize the importance of implementing safety measures in all 3D printing environments, including the home, such as improved ventilation, thermoplastic material, and brand selection. Additionally, our research highlights the need for further regulatory guidelines to ensure the safe use of 3D printing technologies, particularly in the home setting.

## 1. Introduction

Desktop 3D printing is a form of additive manufacturing that allows users to create three-dimensional objects layer by layer from a digital model [1]. This technology has evolved significantly over the years, becoming more accessible and affordable for personal and small-scale use. It can be easily used for prototyping and customization in low volumes and is also used in education to teach children about concepts in STEM, for example. Four-dimensional printing is an extension of three-dimensional printing that adds the dimension of time [2]. The added dimension refers to the ability of a printed object to change its shape or properties over time in response to external stimuli, such as heat, water, or light. This evolution involves the integration of smart materials and programmable elements into the printing process. As well as smart materials, innovation in this field is also being seen in the development of other new printable materials, including ceramic materials, electronic materials, and biomaterials and composites thereof [3]. Although 3D printing is a new enabling technology, there are challenges and complexities around printing with good accuracy, often due to non-uniform thermal gradients during printing that can lead to stress build-up [4]. To minimize this, different materials require different printing conditions for optimal printing.

There are environmental and health considerations that are associated with 3D printing that remain to be fully understood and quantified [5] and mitigation measures put in place. Low-cost 3D printers are growing in use in the domestic setting as well as in maker spaces, schools, and small businesses providing 3D printing services [6]. It is non-industrial settings like these that may carry the highest risk to human health as these printers are typically used in poorly ventilated living spaces, are not operated within enclosures, and usually without PPE. This contrasts with common practice in non-domestic environments (e.g., industry [7]), where printers can be equipped with air quality monitoring capabilities and protective enclosures.

During 3D printing processes, thermoplastic polymer filaments are melted at high temperatures in the printer nozzle to allow extrusion. The thermal decomposition of these polymers process leads to emissions to the surrounding environment of particulate matter (PM) and volatile organic compounds (VOCs), the characteristics of which are dependent on the filament type, the printer type, and print conditions used, e.g., extrusion temperature [8]. Based on numerous exposure studies, the PM emission is significant, likely not benign, and exposure to it should be minimized [9,10,11]. Studies are showing that the VOC emission is also significant, and depending on the polymer filament used, certain characteristic VOCs are emitted, some of which are harmful to human health [12,13].

Among a wide range of thermoplastics used as polymer filaments, acrylonitrile butadiene styrene (ABS) and polylactic acid (PLA) are among the most common. ABS is characterized by high strength, stiffness, and resistance to chemicals. It also requires a higher extruder nozzle and build-plate temperatures than PLA. PLA is biodegradable, thermally unstable, and more brittle compared to ABS [9]. In terms of VOC emissions, ABS printing has been shown in some studies to emit higher amounts of and more diverse VOC profiles [14]. Styrene monomer, a component of ABS starting material, for example, is commonly reported as being emitted in ABS-based 3D printing. Styrene is a suspected human carcinogen. Quantitative studies note that the amount of styrene emitted during 3D printing is relatively low, and the risk of exposure is minimal once proper ventilation is in place. However, if proper ventilation is not in place, as is the case for printers deployed in the home, health risks from exposure to such VOCs increase. PLA-based filament printing has been shown to release a different VOC emission where lactide, methyl methacrylate, and caprolactam are among the most commonly recovered compounds [8,12,14]. Although the principal emission, lactide, is not inherently harmful to human health, PLA is by no means safe for users, particularly when extruded at high temperatures.

As well as the filament thermoplastic character, the composition of the full VOC emission depends on filament brand and color and extrusion temperature used [15] Furthermore, different studies report different VOC profiles because of different analytical workflows implemented. In a recent comprehensive study, Davis reported the emission of >200 VOCs from printing from 5 different filaments, showing the complexity of the emission, with different monomers and thermal degradation by-products unique to specific thermoplastic filaments and print conditions being released.

The present study investigates VOCs and particulate emissions from different ABS and PLA filaments printed with a low-cost, consumer-model home 3D printer to investigate and evaluate the emission profiles from the different filaments and print conditions. VOCs and particulates are sampled from a chamber enclosed by a 3D printer. VOCs are sampled post-printing using a solid-phase microextraction (SPME) method and an untargeted GC–MS method used for their analysis. Particulates are sampled and analyzed in real time during PLA printing with a Wideband Integrated Bioaerosol (WIBS) real-time monitor to provide information on select particle features, including particle size, approximation of particle shape, and fluorescent intensity in 3 detection channels. With VOC profiling and particulate characterization, we aim to give a holistic view of the emission composition from these filaments in the context of evaluating potential health concerns for home users of 3D printers.

## 2. Materials and Methods

### 2.1. 3D Printer and Enclosed Print Chamber

A Creality Ender-3 (www.l3D.ie, accessed on 9 November 2023) fused deposition model desktop home 3D printer was used in this study. The printer is fully motorized and equipped with a single extruder nozzle, heating bedplate (235 mm × 235 mm), active cooling fan, and hot-end fan. The cooling fan is located next to the extruder nozzle and operates constantly at the user-defined speed throughout the length of the print to cool the extruded filament. The hot-end fan is directed onto the motor to maintain temperature.

**Figure 1:**
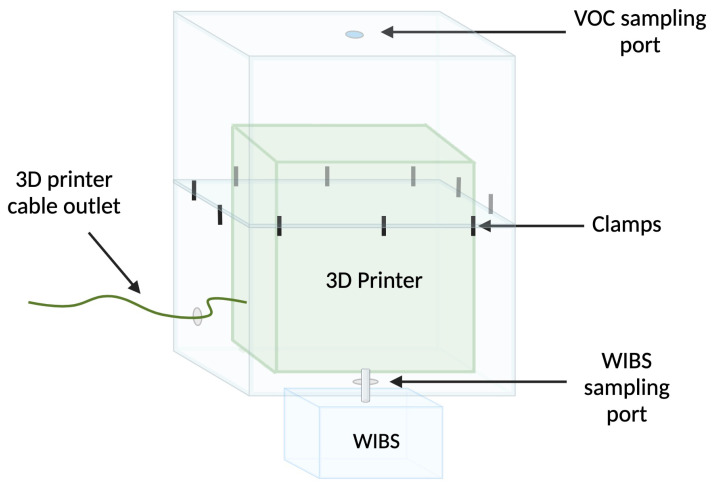
Schematic of printer chamber containing the 3D printer, the VOC sampling, and the particulate sampling ports above and below the printer, respectively (created in Biorender).

Printing was carried out in a homemade printing chamber (Figure 1) comprised of two assembled polytetrafluoroethylene (PTFE) storage containers (Ikea; (Älmhult, Sweden); article number: 901.029.71; box dimensions: 78 cm × 56 cm × 43 cm; box volume 130 L). The printer was placed into one of the containers, and the second was placed on top to seal the chamber. With the printer inside, the containers were clamped together using up to ten retort stand clamps. A rubber septum was fitted to a drilled port in the upper container to allow SPME sampling of the chamber. A hole was also drilled into the bottom of the chamber to allow for monitoring of particulates using the Wideband Integrated Bioaerosol Sensor (WIBS) instrument.

### 2.2. Filaments Used

Various brands and colors of PLA and ABS filaments were tested. All printing filaments were purchased from www.amazon.co.uk (accessed on 9 November 2023). Table 1 gives the colors and brands of the filaments used in the tests.

### 2.3. Printer Settings

A test cube (20 mm × 20 mm × 20 mm) was printed as the standard object for all print runs in this study using the print parameters outlined in Table 2 and Table 3. The gcode file used was created using Ultimaker Cura Software (5.5.0). Pritt stick glue was applied to the printing bed prior to printing to allow for easy removal of the printed cube from the printer bed following printing.

### 2.4. VOC Sampling

VOC sampling was performed using an SPME fiber (divinylbenzene/carboxen/polydimethylsiloxane, 50/30 µm) (Supelco, Bellefonte, PA, USA), which was pre-conditioned for 30 min at 270 °C before use. Immediately after a test cube print from a specified filament, the SPME fiber was inserted into the chamber through the rubber septum (Figure 1) and left for 15 min. Following this, the SPME fiber was retracted, removed, and inserted into the GC–MS for analysis. The chamber was then opened to allow for cooling of the printer and release of VOCs and particulates for a minimum of 40 min. The chamber was reassembled before carrying out another print. Each filament was used to print the test cube and sampled a minimum of three times. Background samples were taken and analyzed daily to establish baseline VOCs and abundances and to identify potential exogenous compounds before printing. For PLA printing, conditions for the background control were that glue was applied to the print bed and that the bed was heated. For ABS printing, no glue was applied, and the bed was not heated.

### 2.5. GC–MS Analysis

Following HS sampling, SPME fibers were injected into an Agilent 7890A series GC connected to an Agilent 5977B mass selective detector (Agilent Technologies, Inc., Santa Clara, CA, USA) for analysis. Separations were performed on an SLB-5ms column (30 m × 0.25 mm, d${}_{f}$ 0.25 μm; Supelco, Bellefonte, PN, USA). Helium was used as the carrier gas at 1 mL/min flow rate. An SPME Merlin Microseal (Merlin Instrument Company, Newark, DE, USA) was installed, and the GC injection port was maintained at a temperature of 260 °C. Splitless injection was used for all samples, and the SPME fiber was desorbed for 4 min within an SPME inlet liner (Supelco). The oven temperature program was isothermal for the first 4 min at 35 °C, then raised to 120 °C at a rate of 5 °C/min, holding for 2 min, then raised again to 270 °C at a rate of 10 °C/min and held for 2 min with a total run time of approx. 40 min. The transfer line to the MS was maintained at 250 °C. The MS was operated with a scan range of 33–330 *m*/*z*, ion source temperature of 230 °C, and an ionizing energy of 70 eV.

### 2.6. WIBS Analysis

Particles produced during the 3D printing of PLA filaments were monitored by the WIBS during printing. The WIBS is a real-time single particle counter that can measure ambient particles between the size ranges of 0.5–30 µm. Full descriptions of the instrument’s inner operation can be seen in previous studies [16]. A basic overview is presented here. The WIBS-4A model was positioned underneath the print chamber with its sampling inlet inserted into the chamber through a port (Figure 1). The WIBS provides information on select particle features, including measurement of individual particle size and approximation of particle shape from the calculation of what is termed asymmetry factors. This is a set value calculated for each recorded particle in the range of 0–100. Typically, a value of 0 would indicate the presence of a perfectly spherical particle, whereas a value of 100 would be representative of a rod-shaped particle. In addition, the WIBS provided details of a particle’s fluorescence in 3 set channels. These channels are commonly referred to as FL1 (excitation: 280 nm, emission: 310–400 nm), FL2 (excitation: 280 nm, emission: 420–650 nm) and FL3 (excitation: 370 nm, emission: 420–650 nm). Depending on whether a particle shows fluorescence in one or more of the set detection bands, the fluorescent fraction sampled by the WIBS can be further subdivided into one of 7 classes—referred to as Perring nomenclature [17] shown in Table S2. The WIBS was originally developed for the detection of bioaerosols. These excitation and emission wavelengths were originally developed for the selective detection of biofluorophores.

Particles were classified as fluorescent if they possessed fluorescence intensity greater than the predefined threshold in at least one of the detection channels. This threshold was calculated by placing the WIBS instrument into forced trigger mode, which is defined as measuring the fluorescence within the optical chamber of the WIBS when it is devoid of particles. Thus, the WIBS fires on empty space and thus allows a baseline fluorescent to be calculated. The baseline in each channel is calculated as the average fluorescence during force trigger mode plus 3 standard deviations.

### 2.7. Data Analysis

The raw chromatographic GC–MS data were analyzed using Agilent MassHunter Acquisition Data Qualitative Analysis version 10.0 software (Agilent Technologies, Santa Clara, CA, USA). Peak acquisition and respective peak areas were determined using a chromatogram deconvolution compound mining algorithm. A peak filter of ≥200,000 abundance counts was set, and only peaks that could be accurately identified and detected in >50% of replicate analyses were included in the final peak list. Background subtraction was carried out by subtracting abundances of compounds recovered from the background chamber samples from compound abundances recovered after 3D printing for the final data set. Identification of compounds and structures was performed using the National Institute of Standards and Technology (NIST) library version 2.3 (2017). Identification of the compounds was supported by their respective mass spectra and retention index (RI) matching with a tolerance of ±15 RI units. A standard mixture of saturated alkanes $(C_{7}$–$C_{30})$ (Sigma Aldrich, Wicklow, Ireland) was run using the same method used for the samples and used for RI matching. In addition to this, confirmation of the retention time (RT) of some compound characteristics to the 3D printing emissions was carried out using commercially available analytical standards (lactide, CAS:95-95-5, purity:99%; caprolactam, CAS: 105-60-2, purity:99%).

Microsoft Excel (version 16.77.1) and RStudio (version 2023.03.0) were used for all data exploration and visualization. VOC and WIBS data analysis was carried out exclusively in R using a myriad of packages, including tidyverse (version 2.0.0; [18]), lubridate (version 1.9.3; [19]) and ggplot2 (v3.3.4; [20]).

## 3. Results and Discussion

### 3.1. VOC Profiling of PLA and ABS Filaments

The extrusion process of thermoplastics generates VOCs, some of which have the potential to cause harm to human health. Hence, during the operation process, it is recommended to use personal protective masks and clothing to minimize exposure, and while this may be the case in industrial environments, it is not the case in the typical home environment. Hence, it is important to assess the profiles of VOC emissions from filaments to understand potential exposure risks for home users. Unfortunately, no standard test method exists for the sampling and analysis of 3D print filaments, so a range of different VOCs and emission profiles have been reported in the literature. In our case, a pre-concentration adsorbent was used to sample the print chamber following the printing of a test cube from each filament. The SPME fiber was then analyzed using GC–MS, as described in the methods section.

**Figure 2:**
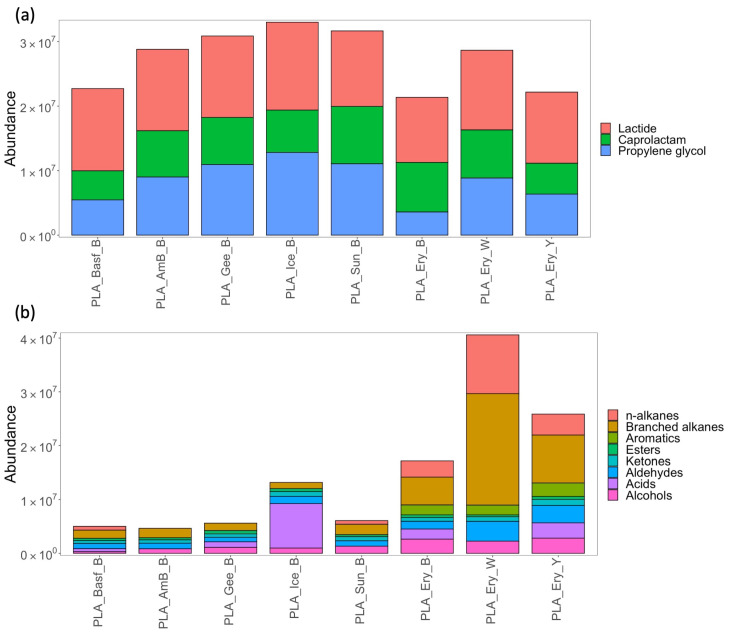
Stacked bar charts showing abundances of (**a**) the most abundant compounds recovered and (**b**) all other identified compounds classified by compound class recovered from different PLA filaments following the printing of test cube (n = 4).

Our workflow to recover and profile VOC print emissions was applied following the printing of a test cube with all PLA and ABS filaments (Table 1). Figure 2 shows the recovered abundances of the five most highly abundant compounds emitted from both PLA (Figure 2a) and ABS (Figure 2b). The key compounds recovered from PLA were lactide, caprolactam, and propylene glycol. Lactide was recovered in the highest abundances across all filaments, and this is consistent with other reports [21]. It is used as the monomer in the ring-opening polymerization synthesis of PLA, and as such, can be present as a residual compound released during melting of the polymer [21]. Caprolactam monomer was recovered in high abundances and has been reported previously as an emission from PLA filaments as well as nylon and ABS filaments. In this case, high abundances were recovered in the control experiments (Supplementary Materials Figure S1) as Pritt Stick glue was applied to the print bed before printing, which releases caprolactam as a VOC. Propylene glycol, the third most abundant compound recovered, is a plasticizer and likely added to PLA filaments to improve processability and flexibility to lower the glass transition temperature and increase ductility [22]. Lactide and caprolactam were reasonably uniform in terms of recovered abundances across the filaments, while propylene glycol was more variable. Although lactide is not listed under major regulatory/health risk lists for indoor air quality, [14] caprolactam is an irritant and is associated with ocular and respiratory toxicity [14]. Methyl methacrylate is another commonly reported VOC to be recovered after PLA printing [14,15] and is a known irritant but was not recovered in this study. The remaining compounds recovered were grouped according to compound class, and their distribution is shown in Figure 2b. It can be seen that while the Ice black filament (PLA_Ice_B) had the highest emission of acids (see Figure S2 for composition), the Eryone black, white and yellow filaments (PLA_Ery_B, PLA_Ery_W, PLA_Ery_Y) had highest recoveries of n-alkanes, branched alkanes and aromatics relative to other filaments. This illustrates the brand-specific nature of the VOC profiles that have also been noted in other works.

**Figure 3:**
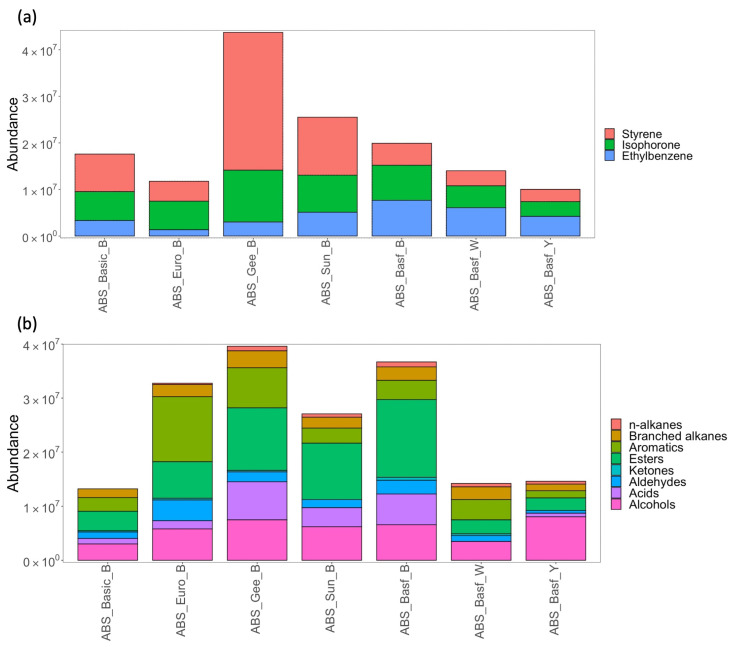
Stacked bar charts showing abundances of (**a**) the most abundant compounds recovered and (**b**) all other identified compounds classified by compound class recovered from the different ABS filaments following the printing of test cube (n = 4).

Figure 3a,b shows equivalent data for ABS-based filament printing. Figure 3a shows the recovered abundances of the three highest emitters–styrene, isophorone, ethylbenzene–for each filament. All these compounds are listed as possible human carcinogens under the IARC and Prop 65 [14]. There is variability in the emissions of all three compounds across the different filaments, notably the elevated abundance of styrene for the black Geeetech filament (ABS_Gee_B), which was the highest emission of any compound across all filaments, recovered in an amount at least double that of any other filament. Given that these compounds are possible human carcinogens, it is of concern that there could be such significant variability in their emission depending on the brand of filament selected by a consumer. Isophorone is a common solvent and was one of the most abundant compounds recovered. This is interesting as no other studies to our knowledge have reported isophorone as a characteristic emission from 3D printer filaments. As with PLA, the remaining compounds recovered were grouped according to compound class (Figure 3b) and again, variability across the n-alkanes, branched alkanes, and aromatics can be observed. For certain brands of black filament (Geeetech and Euroharry), the aromatics were among the highest emissions. In general, the total VOC emissions are greater for ABS compared with PLA.

**Figure 4:**
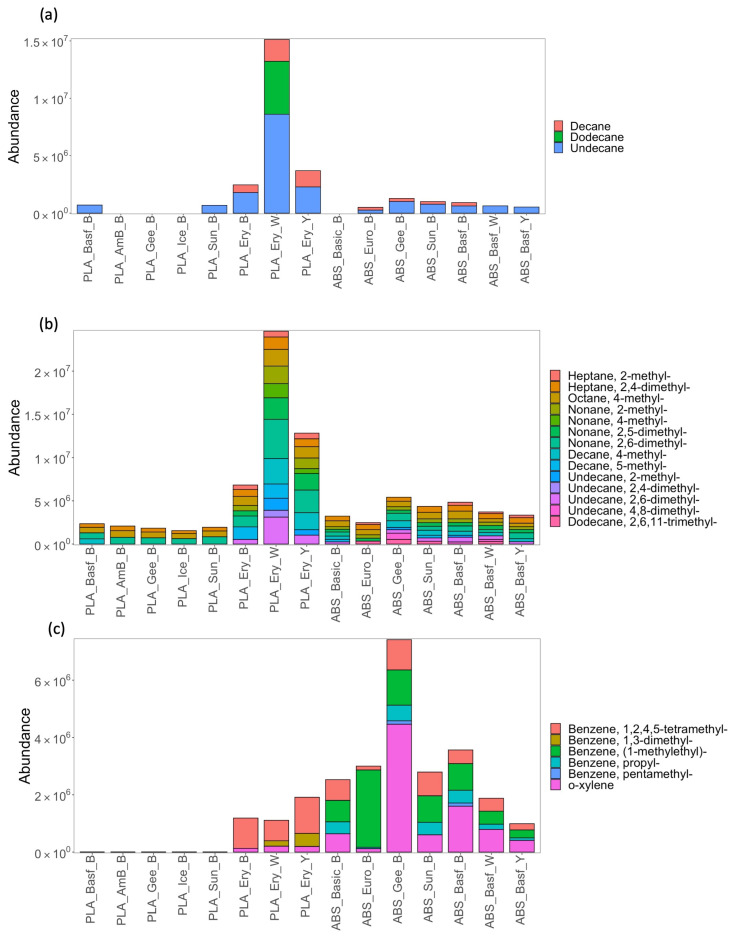
Stacked bar charts showing emission profiles by compound for (**a**) n-alkanes, (**b**) branched alkanes, and (**c**) aromatics for PLA and ABS filaments sampled from the print chamber following the printing of test cube. Compounds already profiled in Figure 2 and Figure 3 are not included here.

**Figure 5:**
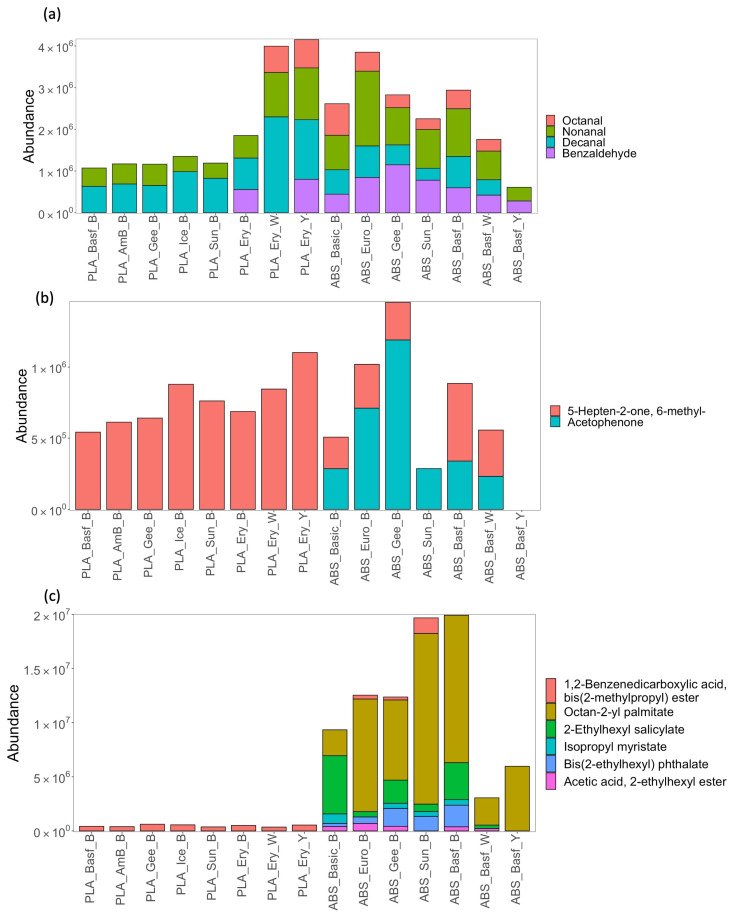
Stacked bar charts showing emission profiles by compound for (**a**) aldehydes, (**b**) ketones, and (**c**) esters for PLA and ABS filaments sampled from the print chamber following the printing of test cube. Compounds already profiled in Figure 2 and Figure 3 are not included here.

Individual compound emission profiles were also examined across all filaments (Figure 4, Figure 5 and Figure S2). Figure 4a–c profiles the emission of identified individual compounds (recovered in abundances greater than threshold) for n-alkanes, branched alkanes and aromatics across all PLA and ABS filaments. Just three n-alkanes were recovered, while branched alkanes were the most diverse compound class recovered. Five aromatic benzene compounds, thermal degradation bi-products of filaments [14], were recovered across all the filaments. One notable trend was the brand specificity of the recoveries across the different compound classes, where all colors of the PLA brand Eryone (PLA_Ery) resulted in significantly higher abundances than all other filaments. This again highlights that the brand of filament used by the consumer has a large impact on the emission profile and, hence, the potential exposure risk to the consumer. Comparing ABS and PLA in terms of overall hydrocarbon profiles (Figure 4), it can be observed that ABS shows the greatest diversity in individual compounds, in particular for the branched alkanes. ABS also has greater recovered abundances of aromatics compared to PLA.

Figure 5 profiles the aldehyde, ketone, and ester emissions for all filaments. Aldehydes octanal, nonanal, and decanal were commonly recovered. These aldehydes are found in indoor air but were recovered in much higher abundances than in control experiments (Figure S1). Benzaldehyde was recovered with some variability from all ABS filaments and just two PLA filaments. It is a degradation product of styrene [23] Two ketones were recovered, whereas acetophenone, another degradation product of styrene, was recovered from ABS only. Benzaldehyde and acetophenone are known irritants [24,25]. Finally, ABS filaments had a diverse and high recovery of esters (Figure 4c) that are potentially present as additives in the filament chemistry.

### 3.2. Particle Analysis

These filaments characterized for their VOC emissions were previously characterized in terms of their particle emissions during 3D printing using an optical particle sizing (OPS) sensor [26]. As semi-volatile organic compounds (SVOCs), including additives, can potentially form particles [27], some VOCs are by-products of these SVOCs that make up particles. Therefore, some relationships between VOCs and particles might be observed [14]. In this case, it was noted that the ABS black BASF filament (ABS_BASF_B) resulted in the highest emissions of particulates. However, in terms of VOCs recovered from this filament, there was no compound or compound class recovered correlating with this high PM emission.

To examine other PM properties, a WIBS instrument was investigated to examine the types of information that could be gathered with this instrumentation, traditionally applied for bioaerosol characterization. Particle emissions from PLA filaments and glue control run (PLA_Con_G) were recorded and characterized in real time by the WIBS. The WIBS has been deployed in a range of different outdoor and indoor campaigns [28,29,30,31,32,33,34,35], and interestingly has been utilized to understand emissions in medical environments, highlighting that human activities and tasks can significantly influence the air quality in indoor spaces [36,37] and that mitigation processes could be of use. This study represents the first use of the WIBS in monitoring particulate emissions released during 3D printing. In total, over 30,000 individual particles were recorded by the WIBS during this investigation.

**Figure 6:**
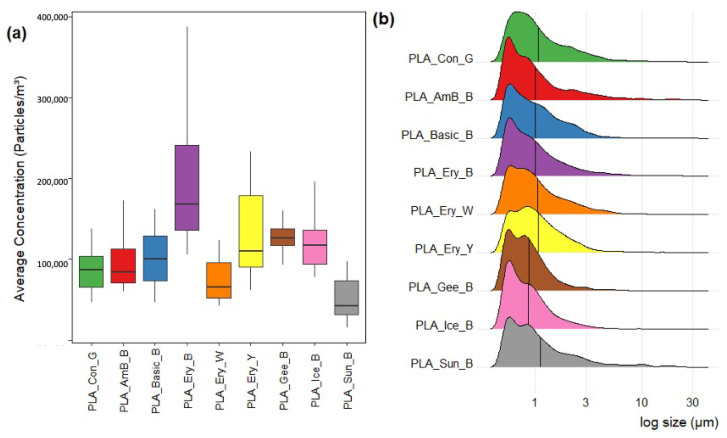
(**a**) Total average minute particle count and (**b**) particle density size distribution (black line illustrates mean particle size and y-axis is density) per filament type.

Particle concentrations were averaged over the number of replicate runs and confined to a minute resolution (Figure 6a). From the examination of the averaged minute particle data, particle concentration produced per minute during printing with the Eryone black filament (PLA_Ery_B) was substantially higher than for any of the other filament types. Similarly, Eryone yellow (PLA_Ery_Y) and Geeetech black (PLA_Gee_B) filaments also produced higher concentrations of particles. Both Eryone white (PLA_Ery_W) and Sunlu black (PLA_Sun_B) filaments produced lower average minute particle concentrations than the control – possibly suggesting the inhibition/masking of particulate matter produced from the glue layer upon coverage by the printed filament layer. These overall trends continued for the evaluation of the total particle concentrations produced for each filament (Table 3). Throughout the printing period, the concentration of particulates peaked within the first 5 min (Figure S4). Following this, a steady decline in particle detection was typically seen for most filament types. This temporal trend has been discussed in detail in our earlier study in Khaki et al. [26], albeit without the use of a print chamber, and can be partly attributed to the reduction in exposed surface area of exuded filament resulting in slower particle emission.

The varying size distributions of particles produced from the different 3D filaments used were also investigated and highlighted in Figure 6b. Most filaments illustrated a maximum particle density at smaller particle sizes. Similar size profiles are apparent, especially for Amazon basics, Basicfil, Eryone, Ice filaments, and Sunlu black filaments. The only exceptions to this trend were seen for Eryone yellow and the control runs. Eryone yellow illustrated a maximum at slightly higher size ranges, whereas the glue control, with no filament use, illustrated a broader shoulder of particle sizes. Former studies have highlighted the importance of particles smaller than 0.5 µm in the ultra-fine particle range [26,38,39]; however, this is below the optical limit of the WIBS. Therefore, the true particle concentrations encountered in this study are anticipated to be similar to those seen in our earlier work [26]. Traditional particle counters commonly used in 3D printing monitoring studies have mainly focused on assessing the size and magnitude of particle emission during complete runs and over temporal periods [12], the WIBS is unique in its capabilities, providing additional information on the shape and fluorescent characteristics of recorded particles. The fluorescent distributions of particles classified for each filament type are further summarized in Table 4.

During monitoring, the majority of particles detected by the WIBS were classified as non-fluorescent (75–91%), with 9–25% of particles possessing fluorescent intensity in at least one of the detection channels above the 3σ threshold. Ice filament black particles illustrated the lowest fluorescent intensity, with 9% of the particles sampled exhibiting fluorescence, whereas Sunlu black (25%), Eryone white (24%), and the control (24%) produced the highest fractions of fluorescent particles. The fluorescent characteristics of PLA-type material have been investigated previously in the literature, showing fluorescence in the 400–500 nm region following excitation in the 300–400 nm region [40] roughly coinciding with the WIBS excitation and emission channels.

Varying degrees of fluorescence WIBS particles were detected during the investigation (classified according to Perring nomenclature), although A and B-type particles dominated the majority. Half of the black filaments produce predominantly A-type particles (33–44% of fluorescent particles). Amazon basics black emissions were dominated by ABC-type particles, whereas glue control ruins were dominated by B-type particles. Eryone branded filaments also produced varying degrees of WIBS-classified particles. Both yellow and white were dominated by A-type particles, while the black filament was dominated by B-type particle emissions. There are several reasons for these variations. For one, the varying brands, as noted for the VOC emissions, vary as they are likely influenced by the presence of various additives, even if the prevailing PLA polymer remains constant. This could result in variations in particle fluorescence and contributing particle types. The relationship between the size of the particle and subsequent WIBS particle classification/fluorescence must also be considered.

Studies have highlighted size-dependent fluorescence of a range of particles classified by the WIBS [41,42]. Typically, as particle size increases, so too does the fluorescence intensity and likelihood of multi-channel fluorescence. This is most apparent for the Amazon black basics filament, which produced the most ABC-type particles and illustrated notable higher distributions of particles at and above 10 μm (Figure S5).

Although this study represents the first use of the WIBS instrument to detect/monitor particle emissions from 3D printing, a preliminary study focusing on the ability of the WIBS to detect nano-plastics also showed similar size dependence on fluorescent classes [43]. This preliminary study largely focuses on common polymeric plastics, such as polyethylene and polypropylene, which possess notable fluorescence and sample larger particle sizes. As a result, the vast majority of particles sampled were seen to possess fluorescence. In comparison, it can be surmised that PLA has a relatively low fluorescence response at the excitation/emission wavelengths used by the WIBS, with larger particles illustrating a higher fluorescence intensity as a direct response to proportionality with size.

Interestingly, notable total and fluorescent particle concentrations were observed for the control. This suggests that the use of glue as a precursor step for 3D printing, in addition to heating the printer bed, also produces particulate emissions. VOC emissions are also observed (Figure S1). The glue (Pritt Stick) used is mainly composed of natural polymers that are not considered to possess fluorescence. Therefore, the fluorescent particles produced could be the result of thermal degradation/processing of glue during bed heating or through PLA remaining on the bed from previous runs. The majority of fluorescent particles were seen at lower size ranges than for filament runs (Figure S5), suggesting that the presence of a portion of smaller fluorescent particles, as well as total particles produced during each run, can be attributed to the use of the glue step.

**Figure 7:**
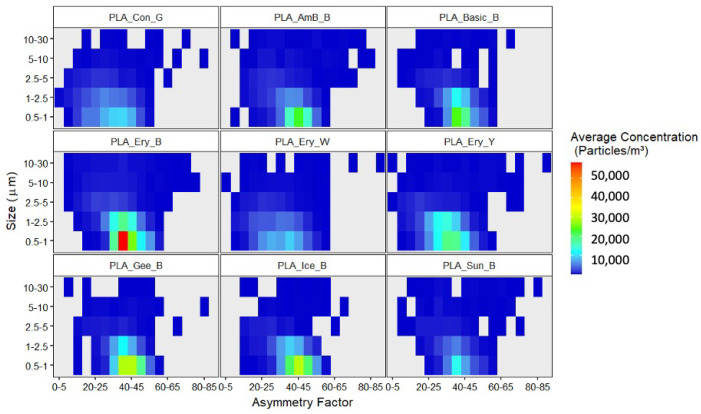
Size vs. AF distribution of all particles detected by the WIBS for the control experiment prior to PLA printing and for the PLA printing of the test cube (26 min).

The shape and size of produced particles were further examined in Figure 7 and Figure S6 for total and fluorescent particles, respectively. One apparent observation is the distinct range in AF seen. For all filament types, particles ranging in sizes from 0.5–30 μm exhibited strong associations with AF values almost exclusively in the range of 20–50, with higher concentrations favoring the 35–50 range. A slight change in size and AF trends were observed for the isolated fluorescent fraction of particles sampled. Fluorescent particles were shown to possess a higher concentration distribution at higher size ranges. This is not unexpected due to the size-dependent nature of fluorescent particles. This is most apparent for the Eryone black filament, which illustrated considerably high concentrations of particle emission in the 0.5–1 μm size range when considering all particles produced. However, upon analysis of the fluorescent fraction, a notable peak in particle concentration at the 1–2.5 μm and 2.5–5 μm is now seen. Similar trends were also noted for many of the other filaments as well as for the control run. The fluorescent particles produced by glue control heat run also exhibit a broader AF range varying from 20–50, illustrating the increased presence of spherical-like and irregular particles. For many of the fluorescent filament particles, the high presence of particles with AF values within the range of 35–50 is still clear; however, filaments that produced higher concentrations of fluorescent PM in the 2.5–5 μm range, such as Amazon basics black, Eryone black, and Eryone white exhibit more diverse ranges in AF values from 15–50. This suggests an increased presence of globular or spherical particles within the fluorescent fraction produced.

These trends in size and AF are very different from what has been seen previously using the WIBS for ambient bioaerosol monitoring, which has been shown to possess a wider variety of AF ranges [30,32]. In contrast, ambient air from a relatively complex environment will illustrate a varying range of overlapping size and AF values indicative of various biogenic and anthropogenic emissions, which is very different from the specific ranges seen here. Previous studies have carried out similar analyses of ambient measurements of particulate matter. In such studies, the detection of fluorescence particles in the outdoor environment is almost exclusively assumed to be due to the presence of bioaerosols or highly fluorescent anthropogenic interferences. Laboratory and field studies using the WIBS-4 for bioaerosol sampling have illustrated that bioaerosols typically result in lower AF values with pollen typically occurring at higher size ranges and AF value less than 25 [16,44] due to many possessing a spherical morphology. Fungal spores, on the other hand, have often shown similar trends in AF ranges as seen for the filament particles, typically occurring within the range of 30–50, albeit at higher size ranges (typically between 2 and 8 μm) [16,44] however certain spores have also favored higher AF ranges of 60 due to several species possessing rod-like morphologies [16,45]. Various WIBS studies can summarize general trends in AF distribution as follows: AF values favoring values up to 20 are indicative of spherical particles, whereas AF values between 30 and 50 AF can be connected to irregularly shaped particles while values above 50 are indicative of rod-shaped particles [32]. Therefore, the particles sampled within the current study can be described as having predominantly irregular shapes, favoring the smaller particle size ranges with fluorescent particle fractions occurring at larger sizes.

## 4. Conclusions

Both VOC and particulate emissions from a 3D printer in a domestic setting were characterized for common thermoplastic filament types on the market. The experimental setup, coupled with an untargeted GC–MS analysis workflow, was used to profile the VOC emissions for PLA- and ABS-based printing and to recover the unique volatiles associated with each filament chemistry. Although filament color was shown to influence emissions as expected, a significant parameter influencing the volatile profile was brand type. Abundances of compounds of concern, such as styrene and ethylbenzene, were, for example, heavily dependent on the brand of filament used.

This study also assessed WIBS instrumentation for characterizing the particulate emissions of PLA filaments where the detection of particles favoring smaller size ranges highlights the importance of establishing and improving occupational safety guidelines, particularly for home use, as exposure to moderate concentration of these particle sizes induces respiratory risks during inhalation. It was not possible to use the current WIBS model to differentiate between different PLA filament brands and colors. Therefore, although no clear differentiation of filament was observed in terms of particulate size distributions and shape for PLA, the ABS filaments would be interesting to assess with this technique, given the fluorescent nature of the base monomers. Further work with the WIBS and other particle counters capable of detecting smaller particle ranges could be used to determine if differentiation between different polymer types is possible.

Although we did not consider rates of the emissions for predicting potential exposure risks to the end-user, this research details the potentially hazardous nature of the emission, further highlighting the need for a standardized method to accurately measure exposure risk when printing with different filaments and conditions, especially in the home setting. This research details the potentially hazardous nature of the 3D printer emission in the domestic and other non-industrial environments for some of the most popular filaments being purchased today. The analysis of other filaments would be of interest to obtain a more holistic picture of the potential impacts on health. We need to be able to measure the emission rates of the VOCs recovered here and all particulates in a standard and comparable way to help inform consumers about exposure risks from different filament types and brands.

## Figures and Tables

**Table 1:** Printing filaments used in this study.

Filament	Color	Brand	Assigned Code
PLA	White	Eryone	PLA_Ery_W
PLA	Yellow	Eryone	PLA_Ery_Y
PLA	Black	Eryone	PLA_Ery_B
PLA	Black	Amazon Basics	PLA_AmB_B
PLA	Black	Sunlu	PLA_Sun_B
PLA	Black	Geeetech	PLA_Gee_B
PLA	Black	Basicfil	PLA_Basic_B
PLA	Black	Ice Filaments	PLA_Ice_B
ABS	White	Basf	ABS_Basf_W
ABS	Yellow	Basf	ABS_Basf_Y
ABS	Black	Basf	ABS_Basf_B
ABS	Black	Sunlu	ABS_Sun_B
ABS	Black	Geeetech	ABS_Gee_B
ABS	Black	Basicfil	ABS_Basic_B
ABS	Black	Ice Filaments	ABS_Ice_B
ABS	Black	Euroharry	ABS_Euro_B

**Table 2:** PLA print settings for test cube printing.

Infill density	20%
Extruder temperature	210 °C
Bed temperature	50 °C
Print speed	50 mm/s
Cooling fan speed	100%
Print run time	26 min

**Table 3:** ABS print settings for test cube printing.

Infill density	20%
Extruder temperature	240 °C
Bed temperature	100 °C
Print speed	50 mm/s
Cooling fan speed	100%
Print run time	33 min

**Table 4:** Overview of particles produced using different PLA filaments.

Filament Type	Total Particle Count Over All Runs	Average Total Particle Concentration	Breakdown of Fluorescent Fractions						
			**A**	**B**	**C**	**AB**	**AC**	**BC**	**ABC**
Control	2966	126,752 particles/m${}^{3}$ Non-fluorescent = 76% Fluorescent = 24%	35%	61%	4%	33%	1%	25%	27%
PLA_AmB_B	3125	133,547 particles/m${}^{3}$ Non-fluorescent = 85% Fluorescent =15%	23%	20%	1%	10%	0%	16%	30%
PLA_Basic_B	3130	133,760 particles/m${}^{3}$ Non-fluorescent = 88% Fluorescent = 12%	16%	37%	1%	6%	0%	18%	11%
PLA_Ery_B	5872	250,940 particles/m${}^{3}$ Non-fluorescent = 82% Fluorescent = 18%	8%	35%	2%	3%	0%	24%	28%
PLA_Ery_W	2484	106,154 particles/m${}^{3}$ Non-fluorescent = 76% Fluorescent = 24%	33%	15%	3%	10%	0%	26%	14%
PLA_Ery_Y	4176	178,462 particles/m${}^{3}$ Non-fluorescent = 87% Fluorescent = 13%	36%	21%	3%	10%	0%	9%	20%
PLA_Gee_B	3576	229,231 particles/m${}^{3}$ Non-fluorescent = 90% Fluorescent = 10%	44%	22%	2%	13%	0%	7%	11%
PLA_Ice_B	3922	167,607 particles/m${}^{3}$ Non-fluorescent = 91% Fluorescent = 9%	33%	31%	2%	15%	0%	6%	12%
ABS_Sun_B	1599	68,333 particles/m${}^{3}$ Non-fluorescent = 75% Fluorescent = 25%	11%	33%	3%	14%	0%	20%	19%

## Data Availability

The data presented in this study are openly available in FigShare at https://doi.org/10.6084/m9.figshare.24535135.v1 (accessed on 10 November 2023).

## Supplementary Materials

The following supporting information can be downloaded at: https://www.mdpi.com/article/10.3390/s23249660/s1, Table S1: Key to codes used in this study matched to those used in earlier particulate matter study on same filaments; Table S2: Seven classes of the fluorescent sub-fractions for the WIBS characterized according to the Perring classifications; Figure S1: Bar charts showing abundances of compounds recovered in control experiments prior to printing for (a) PLA and (b) ABS filaments; Figure S2: Stacked bar charts for (a) alcohols and (b) acids for PLA and ABS filaments sampled from the print chamber following the printing of the test cube; Figure S3: Graph showing the impact of extruder temperature on recovered abundances of compounds within the ABS emission—Styrene, ethylbenzene, isophorone and o-xylene; Figure S4: Time-series of particles detected by the WIBS during PLA printing of the test cube (26 min run time); Figure S5: Ridged density plot (y-axis=density) of the size distribution of fluorescent particles recorded by the WIBS; Figure S6: Size vs AF distribution of (a) fluorescent particles detected by the WIBS and (b) total WIBS classes of fluorescent particles detected by the WIBS during PLA printing of the test cube (26 min run time).

## Abbreviations

The following abbreviations are used in this manuscript:

VOC	Volatile organic compounds
PM	Particulate matter
PLA	Polylactic acid
ABS	Acrylonitrile butadiene styrene
SPME	Solid-phase microextraction
WIBS	Wideband Integrated Bioaerosol
PTFE	Polytetrafluoroethylene
GC–MS	Gas chromatography–mass spectrometry
HS	Headspace
AF	Asymmetry factor
