# Supplementary material for: Characterization of Volatile and Particulate Emissions from Desktop 3D Printers
Source: Sensors (Basel). 2023 Dec 6;23(24):9660. doi: 10.3390/s23249660 (PMC10747962; doi:10.3390/s23249660)
Supplement: Supplementary file 1 [file sensors-23-09660-s001.zip › sensors-2741555-supplementary.pdf]

sensors

MDPI

Article

Supplementary Materials: Characterization of Volatile and Particulate Emissions from Desktop 3D Printers

Melissa Finnegan [1,2], Colleen Lee Thach [3], Shirin Khaki [1,2], Emma Markey [4], David J. O'Connor [1,2], Alan F. Smeaton [2] and Aoife Morrin [1,2,*]

[1] School of Chemical Sciences, National Centre for Sensor Research, Dublin City University, D09 DXA0 Dublin, Ireland; melissa.finnegan22@mail.dcu.ie (M.F.); shirin.khaki2@mail.dcu.ie (S.K.); david.x.oconnor@dcu.ie (D.J.O.)
[2] Insight SFI Research Centre for Data Analytics, Dublin City University, D09 Y5N0 Dublin, Ireland; alan.smeaton@dcu.ie
[3] Department of Chemistry, The University of Kansas, Lawrence, KS 66046, USA; cthach@ku.edu
[4] School of Chemical Sciences, Dublin City University, D09 Y5N0 Dublin, Ireland; emma.markey5@mail.dcu.ie
* Correspondence: aoife.morrin@dcu.ie

check for updates

Citation: Finnegan, M.; Thach, C.L.; Khaki, S.; Markey, E.; O'Connor, D.J.; Smeaton, A.F.; Morrin, A. Supplementary Materials: Characterization of Volatile and Particulate Emissions from Desktop 3D Printers. *Sensors* **2023**, *23*, 9660. https://doi.org/10.3390/s23249660

Academic Editor: Shah Nawaz Burokur

Received: 11 November 2023
Revised: 30 November 2023
Accepted: 4 December 2023
Published: 6 December 2023

Copyright: © 2023 by the authors. Licensee MDPI, Basel, Switzerland. This article is an open access article distributed under the terms and conditions of the Creative Commons Attribution (CC BY) license (https://creativecommons.org/licenses/by/4.0/).

Table S1. Key to codes used in this study matched to those used in an earlier particulate matter study on same filaments.

Filament	Colour	Brand	Assigned Code	Matched Code in [1]
PLA	White	Eryone	PLA_Ery_W	PLAB8w
PLA	Yellow	Eryone	PLA_Ery_Y	PLAB8y
PLA	Black	Eryone	PLA_Ery_B	PLAB8b
PLA	Black	Amazon Basics	PLA_AmB_B	PLAB7b
PLA	Black	Sunlu	PLA_Sun_B	PLAB1b
PLA	Black	Geeetech	PLA_Gee_B	PLAB3b
PLA	Black	Basicfil	PLA_Basic_B	PLAB2b
PLA	Black	Ice Filaments	PLA_Ice_B	PLAB6b
ABS	White	Basf	ABS_Basf_W	ABSB5w
ABS	Yellow	Basf	ABS_Basf_Y	ABSB5y
ABS	Black	Basf	ABS_Basf_B	ABSB5b
ABS	Black	Sunlu	ABS_Sun_B	ABSB1b
ABS	Black	Geeetech	ABS_Gee_B	ABSB3b
ABS	Black	Basicfil	ABS_Basic_B	ABSB2b
ABS	Black	Euroharry	ABS_Euro_B	ABSB4b

Table S2. Seven classes of the fluorescent sub-fractions for the WIBS characterised according to the Perring classifications [2]

Channel	Excitation (nm)	Emission (nm)
A	280	310-400
B	280	420-650
C	370	420-650
AB	280	310-400<br>420-650
AC	280<br>370	310-400<br>420-650
BC	280<br>370	420-650
ABC	280	310-400<br>420-650
	370	420-650

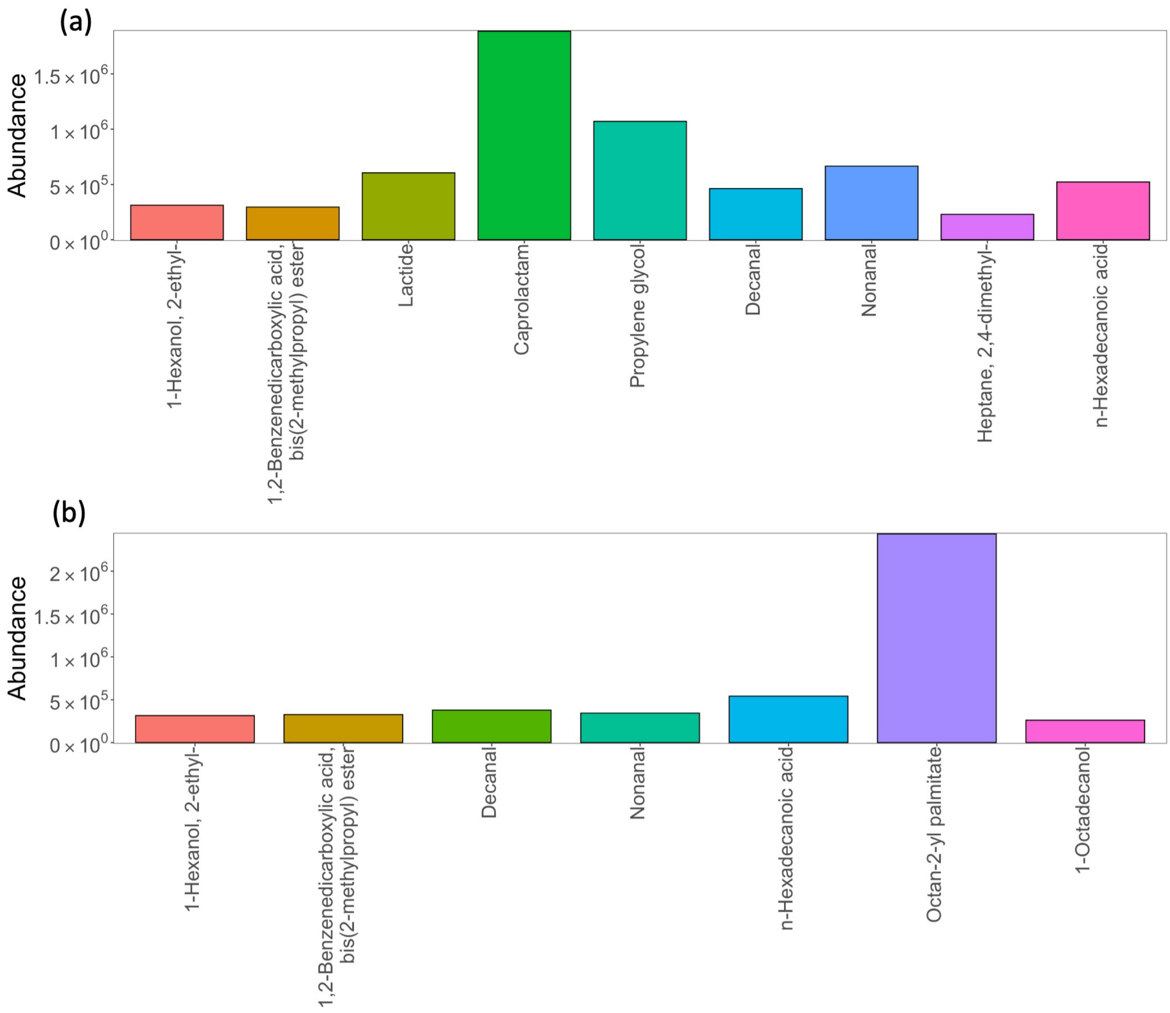

Figure S1. Bar charts showing abundances of compounds recovered in control experiments prior to printing for (a) PLA and (b) ABS filaments. For the PLA control experiments prior to PLA printing, the glue layer was applied and the print bed was heated. For ABS control experiments, glue was not applied and the print bed was not heated. Only compounds recovered for the filaments and were above threshold are included.

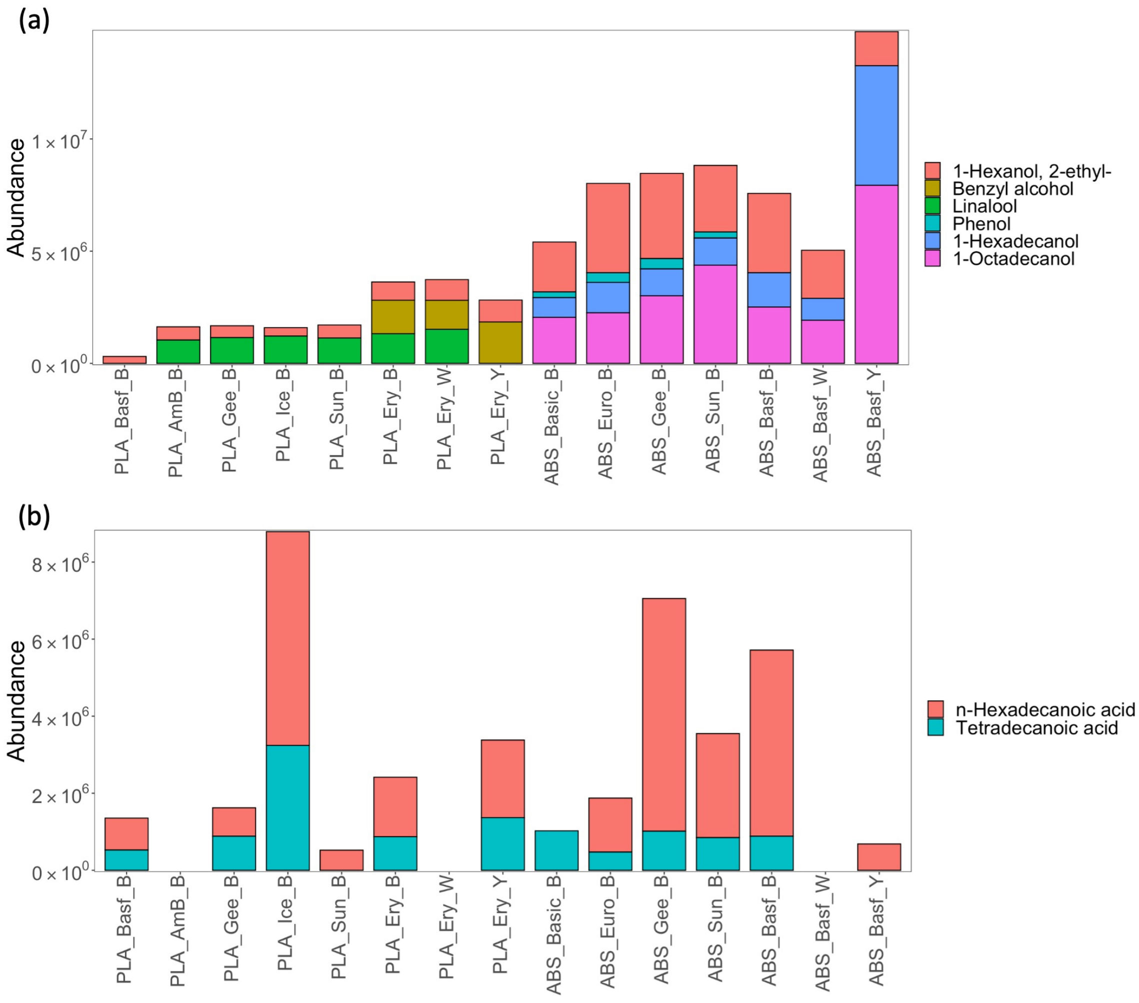

Figure S2. Stacked bar charts for (a) alcohols and (b) acids for PLA and ABS filaments sampled from the print chamber following the printing of the test cube.

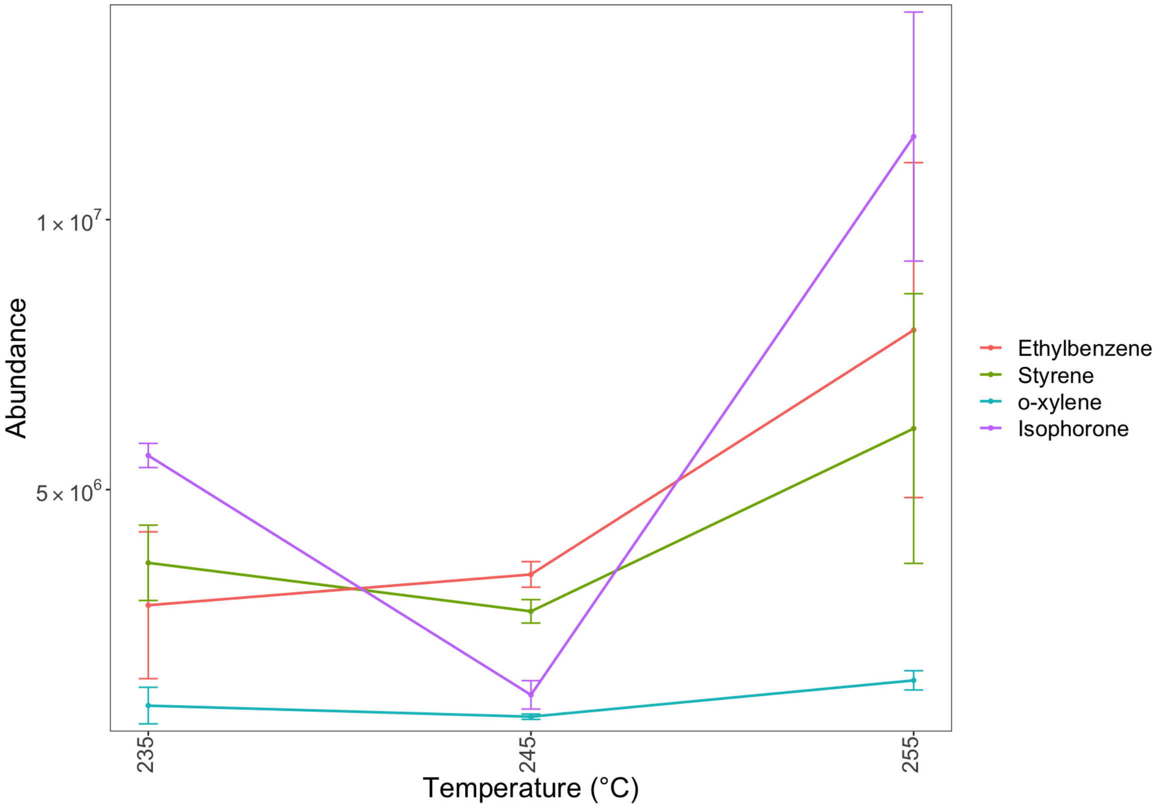

Figure S3. Graph showing the impact of extruder temperature on recovered abundances of compounds within the ABS emission - Styrene, ethylbenzene, isophorone and o-xylene.

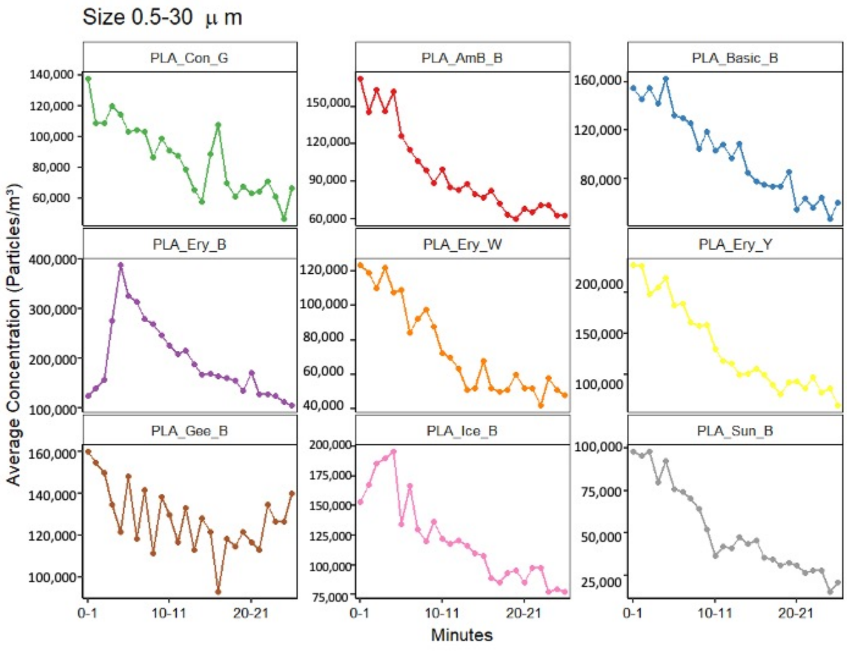

Figure S4. Time-series of particles detected by the WIBS during PLA printing of the test cube (26 min run time).

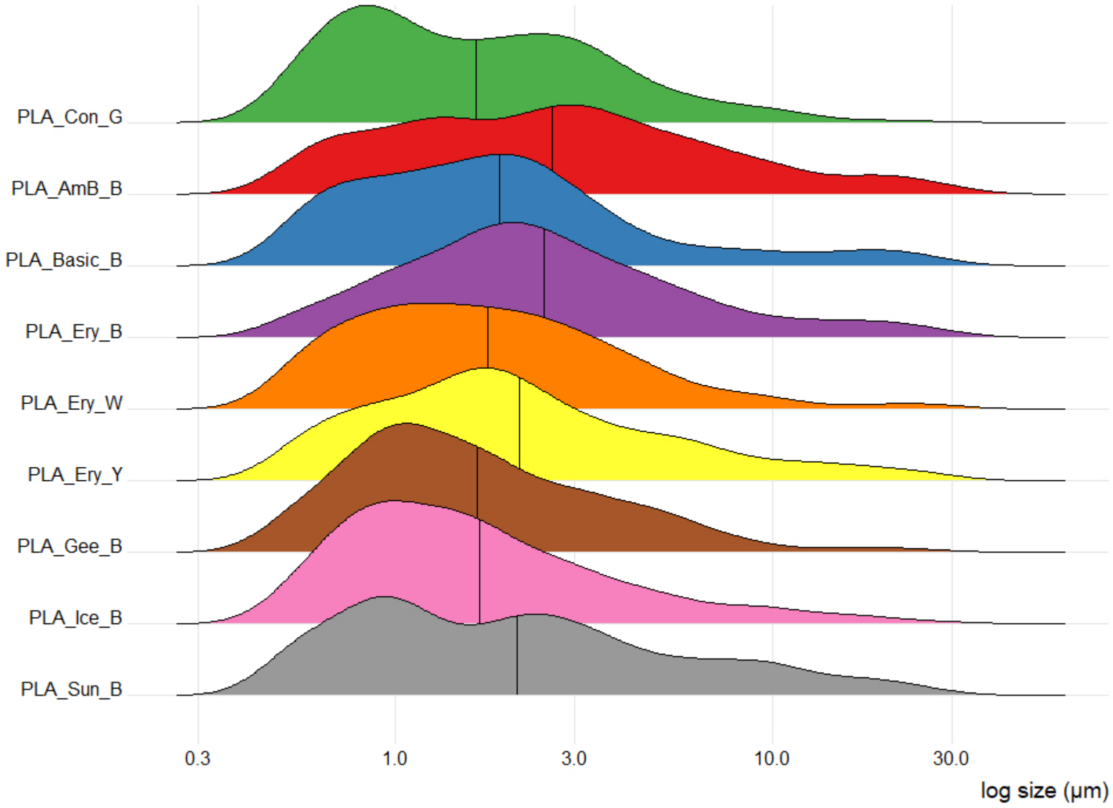

Figure S5. Ridged density plot (y-axis=density) of the size distribution of fluorescent particles recorded by the WIBS.

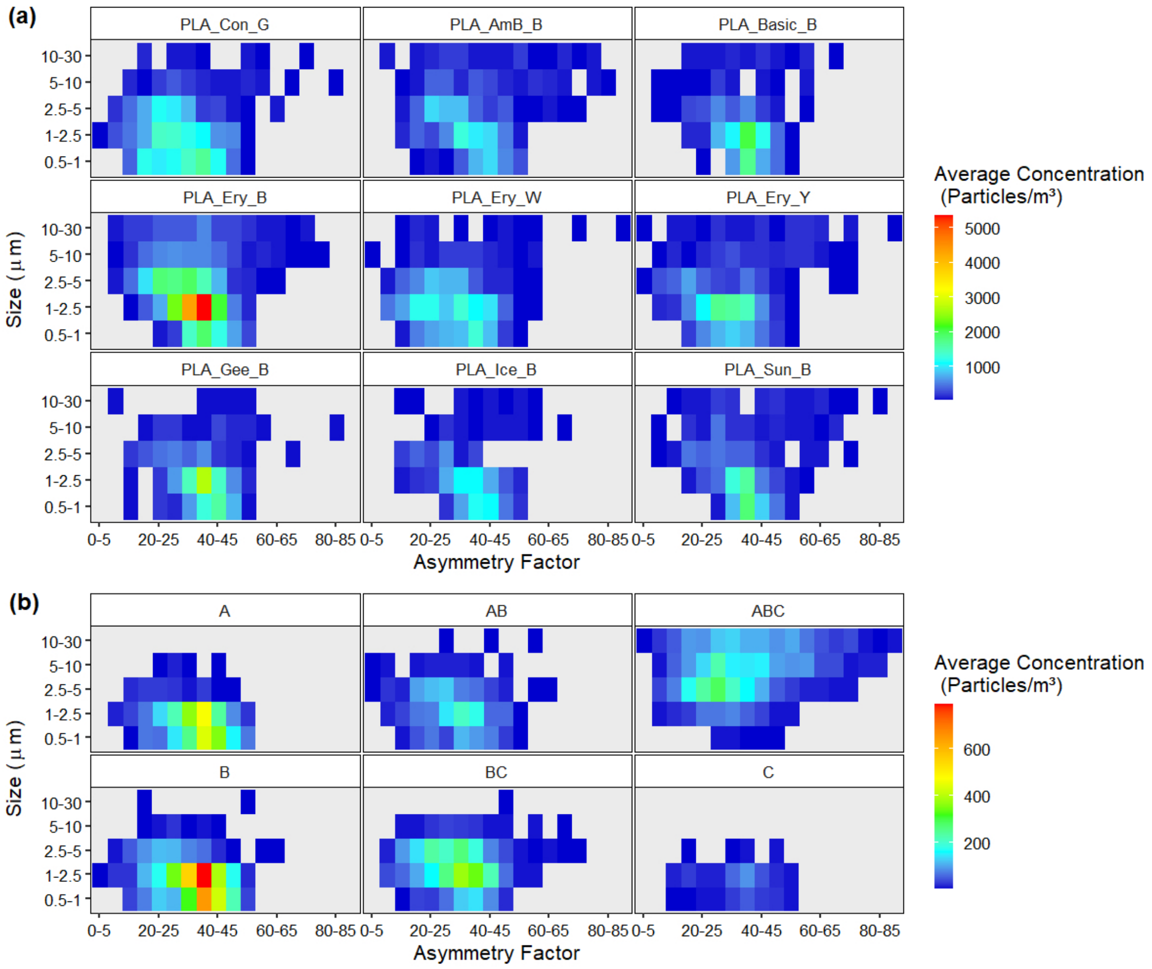

Figure S6. Size vs AF distribution of (a) fluorescent particles detected by the WIBS for each filament and (b) total WIBS-classified fluorescent particles detected by the WIBS during replicate PLA printings of the test cube (26 min run time). Examination of the size and AF distribution of WIBS fluorescent particle classes further corroborates the size dependence of certain classes with smaller size ranges (0.5-2.5 µm) favouring A and B particle classes, slightly larger particles favouring the multi-channel classes such as AB, BC and ABC (2.5-5 μm) and higher concentrations of larger particles almost exclusively favouring the ABC particle class. Larger ABC particles also possessed a wider variety in AF values, illustrating a higher degree in shape variation.

References

1. Khaki, S.; Duffy, E.; Smeaton, A.F.; Morrin, A. Monitoring of Particulate Matter Emissions from 3D Printing Activity in the Home Setting. *Sensors* **2021**, *21*, 3247. https://doi.org/10.3390/s21093247.
2. Perring, A.E.; Schwarz, J.P.; Baumgardner, D.; Hernandez, M.T.; Spracklen, D.V.; Heald, C.L.; Gao, R.S.; Kok, G.; McMeeking, G.R.; McQuaid, J.B.; et al. Airborne observations of regional variation in fluorescent aerosol across the United States. *J. Geophys. Res. Atmos.* **2015**, *120*, 1153–1170. https://doi.org/https://doi.org/10.1002/2014JD022495.

Disclaimer/Publisher's Note: The statements, opinions and data contained in all publications are solely those of the individual author(s) and contributor(s) and not of MDPI and/or the editor(s). MDPI and/or the editor(s) disclaim responsibility for any injury to people or property resulting from any ideas, methods, instructions or products referred to in the content.
